# Cumulative Risks of Foster Care Placement for Danish Children

**DOI:** 10.1371/journal.pone.0109207

**Published:** 2014-10-09

**Authors:** Peter Fallesen, Natalia Emanuel, Christopher Wildeman

**Affiliations:** 1 Rockwool Foundation Research Unit, Copenhagen, Denmark; 2 Department of Sociology, University of Copenhagen, Copenhagen, Denmark; 3 Department of Economics, Yale University, New Haven, Connecticut, United States of America; 4 Department of Policy Analysis and Management, Cornell University, Ithaca, New York, United States of America; University of Louisville, United States of America

## Abstract

Although recent research suggests that the cumulative risk of foster care placement is far higher for American children than originally suspected, little is known about the cumulative risk of foster care placement in other countries, which makes it difficult to gauge the degree to which factor foster care placement is salient in other contexts. In this article, we provide companion estimates to those provided in recent work on the US by using Danish registry data and synthetic cohort life tables to show how high and unequally distributed the cumulative risk of foster care placement is for Danish children. Results suggest that at the beginning of the study period (in 1998) the cumulative risk of foster care placement for Danish children was roughly in line with the risk for American children. Yet, by the end of the study period (2010), the risk had declined to half the risk for American children. Our results also show some variations by parental ethnicity and sex, but these differences are small. Indeed, they appear quite muted relative to racial/ethnic differences in these risks in the United States. Last, though cumulative risks are similar between Danish and American children (especially at the beginning of the study period), the age-specific risk profiles are markedly different, with higher risks for older Danish children than for older American children.

## Introduction

Because foster care placement is such a vital indicator of family instability [1]–[3], it is important to know how common the risk of foster care placement is and whether the risk differs for various groups. Inequality in the risk of foster care placement between groups is especially relevant if foster care harms children, in which case it might exacerbate social inequality, or helps children, in which case it might ameliorate social inequality by improving the lives of the most marginalized children (for studies attempting to isolate causal effects of foster care placement on children, see [4]–[9]).

Recent research [10] estimating the cumulative risk of foster care placement for children in the United States from 2000 to 2011 provides two novel insights into how common and unequally distributed this childhood event is. First, the research shows that foster care placement is far more common than previously thought, with four to five percent of children ever experiencing this event by the age of 18. Second, this event is highly unequally distributed by race/ethnicity, with Native American (about 12 percent) and African American (about 10 percent) children far more likely to ever experience foster care placement before age 18 than Hispanic (about 5 percent), white (about 4 percent), and Asian American (about 2 percent) children. These findings extend previous work that studied the cumulative risk of having a maltreatment report [11], [12], having a confirmed maltreatment case [13]–[15], and the cumulative risk of ever being placed in foster care [16]—all in a US context. The existing literature also found substantial rates of reports, confirmed maltreatment, and foster care entry, as well as pronounced differences across race/ethnicity within the US.

However, these estimates neither provide insight into how high these risks are relative to what we might see in other developed democracies nor show how substantial these racial/ethnic disparities are relative to racial/ethnic disparities in other countries. This makes it difficult to ascertain whether the levels of foster care placement and racial/ethnic inequality in risks of foster care placement in the US are exceptional or not. In this article, we provide one set of estimates against which to compare those generated in the US. As the Danish registry data (which will be discussed below) include complete information on the foster care histories of all children, they provide an excellent opportunity to consider the cumulative risk of foster care placement (using the same methods and roughly the same years) in a comparably developed democracy. We do this by using synthetic cohort life tables to estimate the cumulative risk of foster care placement for children in Denmark by (1) sex and (2) race and parental immigrant status, from 1998–2010.

The Danish case is an intriguing comparison to the US because foster care caseloads have long been rather steady in Denmark [17]. The same cannot be said for foster care caseloads in the United States, which increased dramatically from 1985 to 2000 before dropping thereafter [18], [19]. Point-in-time estimates show that on any given day roughly 1 percent of all Danish children are in foster care [17]. Yet annual caseloads and point-in-time estimates only present an aggregate of foster care cases, and provide little insight into how prevalent foster care placements are as a childhood experience. By estimating the cumulative risks of entering foster care we can unpack to what extent foster care caseloads and point-in-time estimates describe either (a) a small group of children's somewhat constant placements in foster care or (b) a larger group of Danish children who ever experience foster care placement, with some experiencing short stays, others long ones.

### The Danish Foster Care System

The Danish foster care system differs from the US system in a number of noteworthy ways. First, the Danish system relies on a mix of family foster care and institutional care [20], [21], whereas the US places more than three quarters of children in family foster care [5]. Especially older children and children with behavioral disorders tend to enter institutional foster care settings in Denmark (see p. 54 in [21]). Second, the Danish system very rarely relies on non-parental family members to serve as foster parents, whereas this practice is quite common in the US [5], [20], [21]. Finally, the Danish system has historically relied on voluntary and family-oriented interventions [20].

These differences between the Danish and American foster care systems should be kept in mind when comparing the cumulative risks of entering foster care between Denmark and the US. However, recent comparative work has found that across different foster care systems, children who enter foster care exhibit very similar risk factors [22]. So whereas foster care systems differ substantially and children also differ in age at entry (as we will demonstrate in the result section), the children who enter foster care still appear to share risk factors across systems.

## Method

### Data

In order to estimate the cumulative risk of foster care placement for Danish children, we use administrative data on all first time foster care placements from 1998–2010. Municipal Child Services report all initiated child placements. Through unique individual identification numbers, we link foster care information with information on age, gender, and ethnic origin. Thus, the data we use correspond to the data used by [10] because the data include complete information on all children who entered foster care at any point during the study period. For the denominator, we use aggregate data on the full Danish child population, also obtained from Statistics Denmark.

The data we use come from the same registers that caseworkers use to initiate foster care placements. In addition, all Danish citizens are assigned a unique personal identifier at birth or time of immigration. Thus, the data cover all children in Denmark who enter foster care and do not suffer from retention bias, non-response, or other types of selection issues. Moreover, the data linkage does not rely on probabilistic linkage (as is often the case with linked data), because the unique individual identification numbers are constant across years and administrative datasets.

Statistics Denmark anonymizes and de-identifies the data before making it available to researchers. The data can be accessed through any Danish research institution after signing a terms of use agreement with Statistics Denmark. Because the data are retrospective and de-identified, the Yale University Institutional Review Board (where the second and third authors were when this research was performed) deemed this research exempt. Employees of The Rockwool Foundation Research Unit and University of Copenhagen are allowed to publish on the Danish registry data through a longstanding agreement with Statistics Denmark. Whereas the micro data used in this study cannot be made publicly available because of Statistics Denmark's rules regarding protection of privacy, we have made the aggregate data used to generate the synthetic cohort life tables available as supplementary material online (as File S1). Table 1 records the number of children in Denmark who were at risk of experiencing foster care placement at any given age from 1998–2010; Table 2 records the number of children in Denmark who experienced their first foster care placement at any given age from 1998–2010 (see Table S1, Table S2, Table S3, and Table S4 in File S2 for the two tables by gender). First entrance rates decreased across the study period (with only the most modest of decreases in the population of children over this period), and the decrease was most pronounced amongst older children. Yet we need to estimate the changes in cumulative risk to get a clearer picture of the extent of the decrease.

**Table 1:** Number of Children at Risk of First Foster Care Placement in Denmark by Age (0–17) and Year (1998–2010).

	Year												
Age	1998	1999	2000	2001	2002	2003	2004	2005	2006	2007	2008	2009	2010
0	67684	66267	66365	67177	65526	64226	65119	64805	64590	65303	64532	65427	63266
1	68137	67995	66606	66715	67582	65871	64550	65102	65064	64767	65729	64898	65976
2	70825	68329	68158	66815	66904	67702	65884	64600	65157	65072	64980	66068	65203
3	71007	71001	68448	68314	67031	67066	67797	65880	64658	65194	65222	65249	66252
4	68699	71166	71083	68650	68521	67240	67091	67750	65770	64637	65258	65428	65463
5	69268	68837	71257	71201	68885	68706	67279	67060	67677	65758	64710	65429	65543
6	66155	69433	68906	71455	71453	69081	68784	67283	66970	67629	65815	64835	65531
7	65433	66255	69488	69121	71682	71602	69153	68784	67233	66918	67687	65950	64939
8	63395	65665	66357	69661	69307	71853	71714	69227	68736	67178	66938	67760	65981
9	61051	63563	65785	66589	69924	69492	71932	71689	69196	68732	67284	67093	67872
10	58389	61265	63739	66007	66828	70042	69602	71994	71655	69165	68816	67402	67212
11	57808	58559	61392	63940	66257	67063	70169	69687	71966	71674	69272	68986	67535
12	56400	57983	58649	61577	64249	66509	67147	70170	69664	72060	71735	69372	69041
13	54465	56554	58066	58866	61799	64440	66675	67267	70221	69690	72094	71847	69498
14	53370	54696	56735	58323	59148	62049	64587	66765	67256	70298	69793	72334	72022
15	55357	53700	54900	57031	58678	59385	62279	64792	66930	67410	70523	70079	72542
16	55628	55614	53917	55096	57399	58985	59632	62444	64924	67102	67603	70997	70459
17	60194	56208	56182	54606	55844	58056	59652	60284	63010	65459	67636	68230	71290

Source: Statistics Denmark.

**Table 2:** Number of Children Experiencing First Foster Care Placement in Denmark by Age (0–17) and Year (1998–2010).

	Year												
Age	1998	1999	2000	2001	2002	2003	2004	2005	2006	2007	2008	2009	2010
0	157	173	163	192	157	124	143	149	155	165	187	192	144
1	100	96	80	65	65	59	58	59	56	49	52	57	40
2	103	93	78	83	70	46	49	52	54	44	48	49	40
3	112	90	87	94	57	69	58	57	43	59	60	39	40
4	90	102	95	80	82	59	62	45	48	52	43	56	43
5	83	99	92	97	95	63	62	55	60	64	68	45	41
6	104	128	110	100	92	68	67	65	58	56	62	54	45
7	104	135	107	115	100	66	84	75	62	69	79	63	46
8	104	127	131	114	109	78	103	87	71	62	73	70	59
9	112	129	135	141	111	91	82	89	75	79	77	76	45
10	147	156	146	136	110	109	95	100	81	79	80	66	54
11	166	172	172	143	147	134	134	109	88	109	108	92	69
12	163	179	217	187	188	156	156	151	137	159	138	132	70
13	256	244	317	287	274	263	277	280	228	243	244	209	148
14	348	376	426	381	412	357	356	355	301	348	342	329	190
15	399	369	483	435	436	364	378	395	359	385	395	342	219
16	392	383	430	475	459	394	415	424	356	392	374	367	234
17	317	364	402	405	393	355	401	379	303	324	311	288	241

Source: Statistics Denmark.

### Measures of Ethnic Origin

Previous US based work has found large racial/ethnic disparities in the cumulative risk of both confirmed maltreatment and entrance into foster care [10], [14]. Thus, we also examine whether the cumulative risks for foster care placements differ across ethnicity in Denmark. In our definition of ethnicity, it does not matter whether the child was born in Denmark or outside Denmark; ethnic origin depends on the parents' origin. With this in mind, we define three ethnic groups based on parents' country of origin: (1) children born to at least one Danish parent, (2) children born to parents who originate from a Western country (other than Denmark), and (3) children born to parents who originate from a non-Western country. If both parents were born outside Denmark, but one was born in a Western country and the other was born in a non-Western, the mother's ethnic origin defines the child's ethnic origin. Statistics Denmark records ethnic origin for all Danish citizens, immigrants, and descendants of immigrants, so we have information for all children who enter foster care and all children in general.

### Analytic Method

In order to generate estimates of the cumulative risk of foster care placement, we use synthetic cohort life tables, a method originally used to measure mortality in a population [23]. This method uses first-time incidence rates in the population to estimate the proportion of individuals in a hypothetical cohort subject to those age-specific risks at each age that would be placed in foster care by age 18. By converting the rates (_n_m_x_) to probabilities (_n_p_x_), it is possible to create a function of placement for a hypothetical cohort, which can then produce an estimate of how many children could expect to experience foster care placement by age 18. We then use population information on how many individuals are in a given group to determine a cumulative risk figure (_n_c_x_). Life table methods have been widely used in research on the child welfare system in recent years, with some studies adopting only synthetic cohort life table methods [10], [13], [14], some using only birth cohort life table methods [15], and some using both of them to show robustness [16]. For discussions of the comparative benefits and limitations of synthetic cohort and birth cohort life tables, see [10], [16].

Synthetic cohort life tables do, however, have certain limitations. Estimates may be unreliable if the age-specific rates change quickly and the number of synthetic cohorts used is small. Nevertheless, although we do find a substantial decrease in the cumulative risk of foster care placement across the study period, age-specific risks of first entrance decrease gradually and not quickly in our data (with the sole exception being children aged 10 and above in 2010, who did see somewhat large decreases in first entrance rates from the year prior [see Table 2]). Given the large number of years available to us (13), it is also easy to identify years that may have unreliable estimates. In addition, our estimates are similar to birth cohorts estimates found for Danish children born in 1985 [24], indicating both high reliability and robustness (see [16] for discussion of reliability of synthetic cohort estimates when compared to birth cohort estimates).

### Comparing with Estimates for the United States

Our estimates of cumulative risks of foster care in Denmark are to a very large extent comparable to the estimates for the United States provided by [10], with one important difference. Whereas [10] use race-, age-, sex- and year-specific population estimates from [25] as the denominator for number at children at risk of entering foster care, we instead rely on full population registers containing demographic information on all individuals living in Denmark January 1 for each year in our study period. Relying on actual population data instead of population estimates makes the estimates for Denmark somewhat less uncertain than those provided by [10]. Yet, the US Census Bureau estimates are still highly accurate when using groups of sizes above county-level [25] and any inaccuracies will only affect the denominator of our analysis, so we expect differences in accuracy to be negligible. The Danish and American data used to calculate the numerator (children entering into care for the first time) are drawn from official registers containing all children in foster care in both countries [26]–[29]. Thus, these data are judged to be highly reliable and comparable.

## Results

We start by presenting a partial life table for the year 2005 in Table 3 which shows how the cumulative risk of foster care placement accumulates between birth and age 18 for all children in Denmark, native Danish children, Western children, and non-Western children in a sample year. According to the results shown in Table 3, 4.5 percent of all Danish children can expect to ever experience foster care placement at some point in their childhood based on the 2005 first-time admission rates. This is somewhat lower than what is observed in the United States for the same year ([10] reports a risk of 5.9 percent). Both Western children and Non-western children had higher cumulative risks at age 17 than native Danish children, although these differences were not nearly as marked as in the US. As the table also indicates, first-time admissions to foster care are high shortly following the birth and then trail off until the teenage years, at which point they spike dramatically–a finding consistent with trends found in the United States [19]. This teenage spike is, however, especially noteworthy since it is so much greater than the spike for American children [10], suggesting differences in the timing of admissions between Denmark and the US.

**Table 3:** Cumulative Risks of Foster Care Placement from Birth to Age 18 for All Children in Denmark and Native Danish Children, Western Children, and Non-Western Children, 2005.

Age	All Danish Children	Native Danish Children	Western Children	Non-Western Children
0	0.002	0.002	0.001	0.003
1	0.003	0.003	0.001	0.003
2	0.004	0.004	0.001	0.005
3	0.005	0.005	0.001	0.007
4	0.006	0.005	0.001	0.009
5	0.006	0.006	0.003	0.010
6	0.007	0.007	0.004	0.011
7	0.008	0.008	0.009	0.012
8	0.010	0.009	0.009	0.013
9	0.011	0.010	0.010	0.016
10	0.012	0.012	0.016	0.017
11	0.014	0.013	0.017	0.019
12	0.016	0.015	0.020	0.022
13	0.020	0.019	0.032	0.026
14	0.025	0.025	0.041	0.032
15	0.032	0.030	0.050	0.043
16	0.038	0.037	0.055	0.054
17	0.045	0.042	0.062	0.065

Source: Own calculation on data from Statistics Denmark.

**Figure 1:**
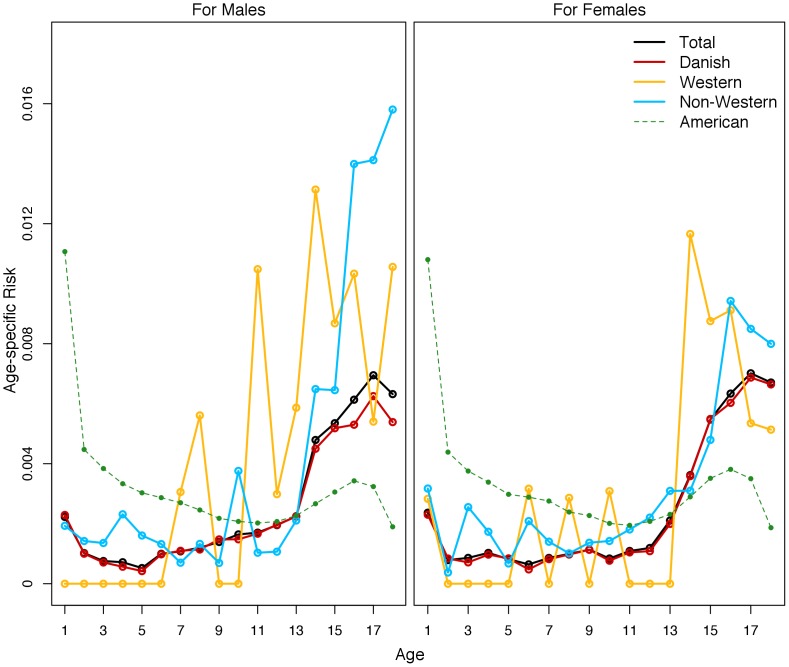
Age-specific Risk of First-time Foster Care Placement in 2005 in Denmark, all Children by Sex and Ethnicity.

Figure 1 further demonstrates just how large this spike is in Denmark relative to the US by showing the age-specific risks for foster care placement across age, gender, and ethnicity. Both boys and girls had high risks of first foster care placement during infancy, then the risk of first placement trailed off until age 13, at which point it increased throughout adolescence to reach its peak around age 16 (although, again, this was the peak only in Denmark, as the US peak occurred in the first year of life, as Figure 1 clearly indicates). When breaking the age-patterning of first placement down by ethnic origin, we see that native Danish children of both sexes and non-Western girl children had similar patterns. In contrast, both Western boys and girls and non-Western boys had different patterns. Both Western groups are numerically small, which explains their less clear pattern. Non-Western boys, however, had increasing age-specific risk across age, with more than 4.4 percent of all non-Western boys entering foster care for the first time after age 14.

**Figure 2:**
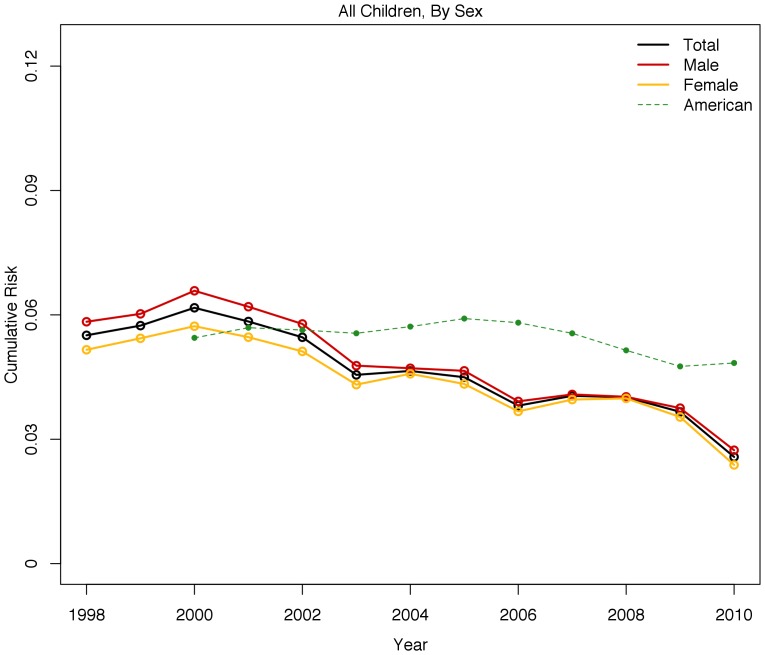
Cumulative Risk of Foster Care Placement by Age 18 in Denmark, all Children by Sex.

Figure 2 shows the trend from 1998 to 2010 in the cumulative risk of foster care placement for male and female children in Denmark relative to the US. The cumulative risk of foster care placement increases slightly until 2000, at which point it then declines until 2005. The cumulative risk remains roughly constant from there until 2008, after which it declines again. Boys have slightly higher risks in the late 1990s and early 2000s than girls have. The sex difference in the cumulative risk of foster care placement disappears in the later 2000s and is relatively small until then. Furthermore, the decline in the cumulative risk of foster care placement within Denmark is so dramatic toward the end of the period that by 2010, Danish children had lower cumulative risks of placement than American children had even though their risk early in the period far exceeded the risk for American children.

**Figure 3:**
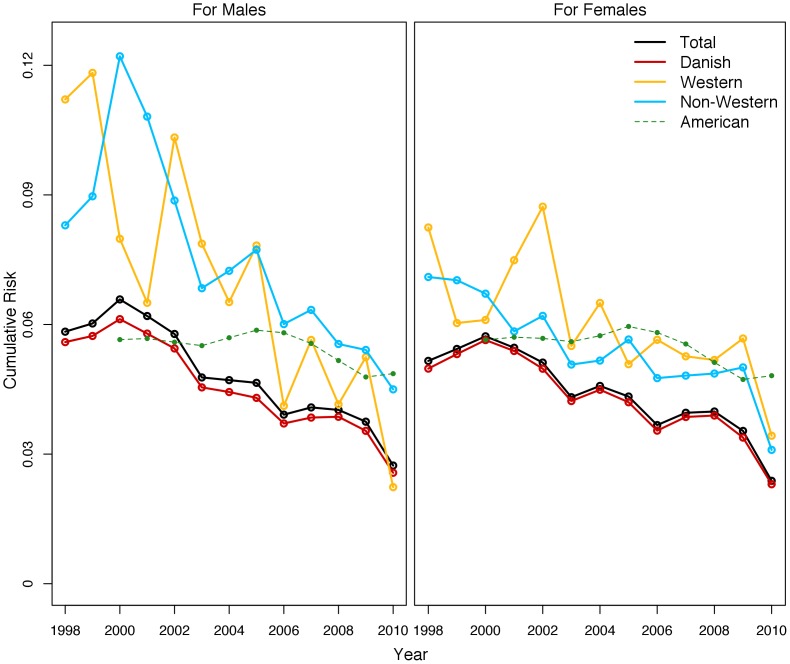
Cumulative Risk of Foster Care Placement by Age 18 in Denmark, by Sex and Ethnicity.

Figure 3 shows results for the total population, as well as for native Danes, Western immigrants, and non-Western immigrants by sex relative to the US. Although Danish children have lower cumulative risks than do other children, these differences are fairly small—especially when compared to the racial/ethnic disparities shown in the US [10]. Depending on the year, cumulative risks for Western and non-Western immigrants were anywhere from slightly above those of native Danes to about double the risk for native Danes. By 2010, however, cumulative risks are only slightly higher for immigrants than they were for native Danes, with more pronounced disparities for males than for females. Girls of non-Western backgrounds have lower cumulative risks than do girls of Western backgrounds for most years. The reverse is true for boys. Comparing across sexes, we find girls overall to have a somewhat lower risk of placement. For Western and non-Western girls, the cumulative risk starts lower than that of boys of the same ethnic origin. The cumulative risk of placement for non-Western girls remains lower than that for non-Western boys, although the risk for Western boys plummets to below that of Western girls in the later 2000s.

**Figure 4:**
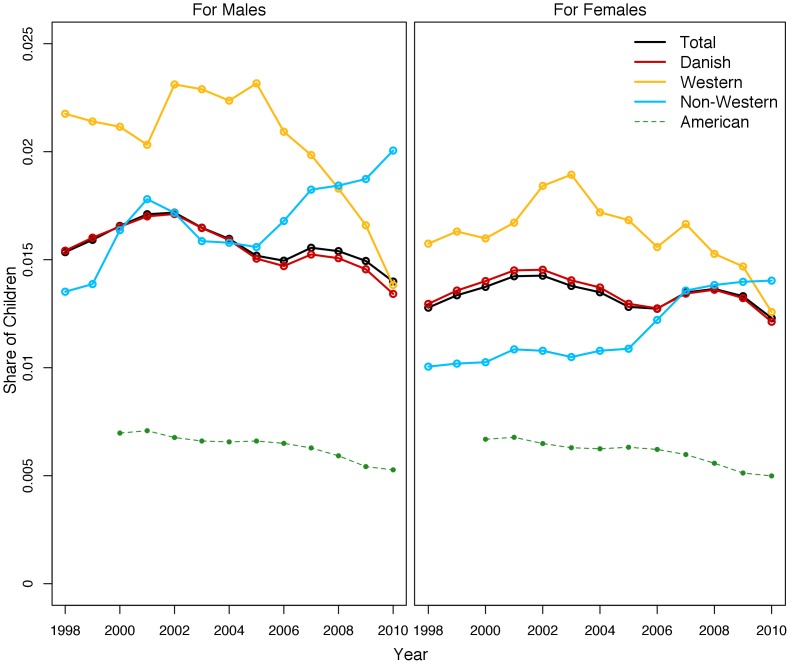
Share of Children in Foster Care at December 31^st^ each Year in Denmark, by Sex and Ethnicity.

The share of children in care at the end of each year, as shown in Figure 4, does not decline as the cumulative risk does, however. Danish girls retain a constant placement share at around 1.5 percent; Danish boys show a trend of limited decline. The placement shares for non-Western children increase across the period. The share of Western boys in foster care declines dramatically in the second part of the 2000s while the share for Western girls remains rather constant, though with a slight positive bump in the first part of the 2000s and a decline in the end of the 2000s.

The fact that the share of children in foster care remains roughly constant throughout the study period while cumulative risk estimates are declining suggests that while intake levels may have decreased over the study period, the length of stay is probably increasing.

## Discussion

Recent research on the cumulative risk of foster care placement in the US provided support for two conclusions [10]. First, far more children will ever be placed in foster care by age 18 than most scholars of American family life would have suspected, with between four and five percent of American children ever experiencing this event by the time they turn 18. Second, this risk is unequally distributed, with up to 15 percent of Native American children and as little as 2 percent of Asian American children ever experiencing this event by age 18 (depending on the year), suggesting potentially important implications for inequality (provided placement has any effects).

The goal of this article was to provide context for these results by examining other developed democracies, using Denmark as a test case. If the Danish estimates roughly correspond with the US results, then that would suggest that the foster care system might merit much more attention from family demographers and scholars of social inequality across the world than it tends to receive. But if the US estimates were dramatically higher than the Danish ones, that might suggest that the foster care system merits greater attention in the US but relatively little attention outside of it. The Danish results we supply here provide tentative support for the idea that foster care is far more common than often thought not just in the US, but also in Denmark, as the cumulative risk of foster care placement at the beginning of the period (in 1998), at around six percent, was higher than estimates over in the same period in the US.

The Danish estimates differed markedly from the earlier American estimates in three key regards. First, the cumulative risk of foster care placement for all Danes plummeted between 1998 and 2010, leading to far lower cumulative risks of foster care placement by the end of the period, with Danish children experiencing a risk of about three percent relative to the five percent risk faced by American children. Second, inequality in the cumulative risk of foster care placement was significantly smaller in Denmark than in the US, suggesting that if foster care placement matters for inequality, it likely does so in the US but not in other developed democracies—or at least not Denmark (or that it matters more for class inequality more than for racial/ethnic inequality, a key alternative to consider). Third, the age-specific risk of first entrance into foster care for Danish children was highest for teenagers, whereas the risk in the US is highest for very youngest children.

A number of explanations for why the cumulative risk of foster care in Denmark decreased in the period 1998–2010 offer themselves. First, one would expect that the financial crisis could have affected the municipal economy, such that there was less budget-room for placing children in foster care in the end of the study-period. However, given that retention rates of children already in foster care were stabile while entrance rates dropped, this does not appear likely. A second possibility is that the study period saw changes in thresholds at which social workers deemed a foster care placement necessary. Some reforms did that take place over the period [20], but their content was not such that we should expect substantial changes in foster care take up.

A third option relates to changes in demand for foster care placement driven by changes in child and parental characteristics. Recent work [30] has shown that a Danish penal reform that lowered the risk of experiencing paternal incarceration led to lower risk of foster care entry for children. Children's characteristics might also have changed during the study period. Children who enter foster care generally displays more behavioral problems that other children, for example ADHD [31]. Previous work has linked untreated ADHD to increased family strain [32] and has also shown that Danish medical treatment rates for ADHD soared in our study period [33]. If medical treatment of ADHD lowers family strain it might also lower the risk of maltreatment, thereby leading to lower risk of foster care entrance—this could be a fruitful avenue for future work.

## Supporting Information

File S1 **Data Used to Construct Life Tables.**
(ZIP)

File S2 **Number of Children at Risk of First Foster Care Placement and Experiencing First Foster Care Placement in Denmark by Age (0-17), Year (1998-2010), and gender.**
(DOCX)
