# Supplementary material for: Cumulative Risks of Foster Care Placement for Danish Children
Source: PLoS One. 2014 Oct 9;9(10):e109207. doi: 10.1371/journal.pone.0109207 (PMC4192300; doi:10.1371/journal.pone.0109207)
Supplement: File S2 — Number of Children at Risk of First Foster Care Placement and Experiencing First Foster Care Placement in Denmark by Age (0-17), Year (1998-2010), and gender. (DOCX) [file pone.0109207.s002.docx]

**Table S1: Number of Boys at Risk of First Foster Care Placement in Denmark by Age (0-17) and Year (1998-2010)**

|  | Year | | | | | | | | | | | | |
| --- | --- | --- | --- | --- | --- | --- | --- | --- | --- | --- | --- | --- | --- |
| Age | 1998 | 1999 | 2000 | 2001 | 2002 | 2003 | 2004 | 2005 | 2006 | 2007 | 2008 | 2009 | 2010 |
| 0 | 34803 | 34098 | 33942 | 34459 | 33510 | 33014 | 33390 | 33172 | 32952 | 33587 | 33033 | 33712 | 32481 |
| 1 | 35002 | 34921 | 34244 | 34082 | 34587 | 33651 | 33145 | 33337 | 33272 | 33013 | 33802 | 33215 | 33985 |
| 2 | 36389 | 35091 | 34978 | 34338 | 34166 | 34629 | 33639 | 33156 | 33342 | 33247 | 33122 | 33941 | 33376 |
| 3 | 36283 | 36480 | 35165 | 35050 | 34431 | 34240 | 34671 | 33632 | 33197 | 33364 | 33282 | 33256 | 34038 |
| 4 | 35202 | 36377 | 36529 | 35260 | 35152 | 34495 | 34252 | 34618 | 33568 | 33219 | 33390 | 33420 | 33343 |
| 5 | 35584 | 35277 | 36432 | 36585 | 35376 | 35275 | 34493 | 34262 | 34584 | 33564 | 33267 | 33507 | 33461 |
| 6 | 33915 | 35683 | 35313 | 36575 | 36718 | 35469 | 35312 | 34498 | 34195 | 34568 | 33612 | 33325 | 33555 |
| 7 | 33571 | 33973 | 35727 | 35443 | 36685 | 36802 | 35509 | 35302 | 34499 | 34183 | 34594 | 33662 | 33371 |
| 8 | 32468 | 33709 | 34025 | 35808 | 35541 | 36782 | 36864 | 35493 | 35264 | 34472 | 34173 | 34620 | 33670 |
| 9 | 31410 | 32573 | 33768 | 34134 | 35941 | 35632 | 36786 | 36845 | 35508 | 35262 | 34531 | 34253 | 34691 |
| 10 | 30089 | 31493 | 32641 | 33873 | 34259 | 36003 | 35694 | 36820 | 36838 | 35506 | 35331 | 34581 | 34288 |
| 11 | 29650 | 30177 | 31575 | 32756 | 34007 | 34397 | 36082 | 35745 | 36813 | 36836 | 35540 | 35382 | 34644 |
| 12 | 28767 | 29747 | 30233 | 31676 | 32936 | 34172 | 34439 | 36082 | 35732 | 36849 | 36909 | 35613 | 35432 |
| 13 | 27843 | 28868 | 29774 | 30354 | 31808 | 33036 | 34254 | 34519 | 36101 | 35750 | 36859 | 36967 | 35676 |
| 14 | 27213 | 27967 | 28965 | 29921 | 30473 | 31935 | 33150 | 34294 | 34497 | 36173 | 35803 | 36950 | 37042 |
| 15 | 28389 | 27373 | 28054 | 29119 | 30128 | 30617 | 32068 | 33230 | 34372 | 34553 | 36258 | 35935 | 37063 |
| 16 | 28272 | 28501 | 27477 | 28144 | 29301 | 30292 | 30723 | 32153 | 33286 | 34483 | 34639 | 36491 | 36156 |
| 17 | 30686 | 28568 | 28800 | 27791 | 28518 | 29573 | 30588 | 31013 | 32419 | 33529 | 34749 | 34948 | 36645 |

Source: Own calculation on data from Statistics Denmark.

**Table S2: Number of Girls at Risk of First Foster Care Placement in Denmark by Age (0-17) and Year (1998-2010)**

|  | Year | | | | | | | | | | | | |
| --- | --- | --- | --- | --- | --- | --- | --- | --- | --- | --- | --- | --- | --- |
| Age | 1998 | 1999 | 2000 | 2001 | 2002 | 2003 | 2004 | 2005 | 2006 | 2007 | 2008 | 2009 | 2010 |
| 0 | 32881 | 32169 | 32423 | 32718 | 32016 | 31212 | 31729 | 31633 | 31636 | 31715 | 31496 | 31712 | 30782 |
| 1 | 33135 | 33074 | 32362 | 32633 | 32995 | 32220 | 31405 | 31765 | 31790 | 31754 | 31926 | 31683 | 31991 |
| 2 | 34436 | 33238 | 33180 | 32477 | 32738 | 33073 | 32245 | 31444 | 31815 | 31825 | 31858 | 32126 | 31827 |
| 3 | 34724 | 34521 | 33283 | 33264 | 32600 | 32826 | 33126 | 32248 | 31461 | 31830 | 31939 | 31993 | 32213 |
| 4 | 33497 | 34789 | 34554 | 33390 | 33369 | 32745 | 32839 | 33132 | 32202 | 31418 | 31868 | 32008 | 32120 |
| 5 | 33684 | 33560 | 34825 | 34616 | 33509 | 33431 | 32786 | 32798 | 33091 | 32192 | 31443 | 31922 | 32082 |
| 6 | 32240 | 33750 | 33593 | 34880 | 34735 | 33612 | 33472 | 32785 | 32774 | 33061 | 32203 | 31510 | 31976 |
| 7 | 31862 | 32282 | 33761 | 33678 | 34997 | 34800 | 33644 | 33482 | 32733 | 32734 | 33093 | 32288 | 31568 |
| 8 | 30927 | 31956 | 32332 | 33853 | 33766 | 35071 | 34850 | 33734 | 33472 | 32706 | 32764 | 33139 | 32311 |
| 9 | 29641 | 30990 | 32017 | 32455 | 33983 | 33860 | 35146 | 34844 | 33687 | 33467 | 32753 | 32840 | 33181 |
| 10 | 28300 | 29772 | 31098 | 32134 | 32569 | 34039 | 33908 | 35174 | 34817 | 33659 | 33485 | 32820 | 32923 |
| 11 | 28158 | 28382 | 29817 | 31184 | 32250 | 32666 | 34087 | 33942 | 35153 | 34836 | 33732 | 33604 | 32891 |
| 12 | 27633 | 28235 | 28416 | 29901 | 31313 | 32337 | 32708 | 34088 | 33932 | 35209 | 34826 | 33759 | 33609 |
| 13 | 26622 | 27686 | 28291 | 28512 | 29991 | 31404 | 32421 | 32748 | 34120 | 33940 | 35235 | 34880 | 33822 |
| 14 | 26157 | 26728 | 27770 | 28402 | 28675 | 30114 | 31437 | 32471 | 32759 | 34122 | 33990 | 35384 | 34979 |
| 15 | 26967 | 26327 | 26845 | 27912 | 28550 | 28768 | 30211 | 31562 | 32557 | 32857 | 34265 | 34143 | 35479 |
| 16 | 27356 | 27113 | 26439 | 26952 | 28098 | 28693 | 28909 | 30291 | 31638 | 32616 | 32964 | 34504 | 34302 |
| 17 | 29508 | 27640 | 27382 | 26815 | 27326 | 28483 | 29064 | 29271 | 30591 | 31929 | 32887 | 33281 | 34645 |

Source: Own calculation on data from Statistics Denmark.

**Table S3. Number of Boys Experiencing First Foster Care Placement in Denmark by Age (0-17) and Year (1998-2010).**

|  | Year | | | | | | | | | | | | |
| --- | --- | --- | --- | --- | --- | --- | --- | --- | --- | --- | --- | --- | --- |
| Age | 1998 | 1999 | 2000 | 2001 | 2002 | 2003 | 2004 | 2005 | 2006 | 2007 | 2008 | 2009 | 2010 |
| 0 | 83 | 82 | 81 | 110 | 81 | 59 | 77 | 74 | 80 | 79 | 92 | 89 | 77 |
| 1 | 50 | 52 | 37 | 34 | 27 | 29 | 23 | 34 | 31 | 22 | 22 | 27 | 24 |
| 2 | 51 | 48 | 36 | 50 | 37 | 26 | 29 | 25 | 38 | 28 | 27 | 25 | 20 |
| 3 | 47 | 45 | 45 | 49 | 28 | 36 | 24 | 24 | 24 | 35 | 27 | 22 | 26 |
| 4 | 45 | 61 | 51 | 48 | 45 | 35 | 32 | 18 | 32 | 31 | 21 | 27 | 26 |
| 5 | 51 | 53 | 57 | 55 | 47 | 41 | 27 | 34 | 30 | 31 | 44 | 30 | 25 |
| 6 | 53 | 75 | 57 | 71 | 54 | 38 | 39 | 37 | 40 | 23 | 39 | 30 | 25 |
| 7 | 58 | 77 | 67 | 60 | 59 | 38 | 43 | 42 | 30 | 41 | 42 | 37 | 21 |
| 8 | 56 | 74 | 87 | 68 | 64 | 48 | 64 | 49 | 41 | 35 | 43 | 39 | 38 |
| 9 | 74 | 82 | 84 | 102 | 66 | 56 | 48 | 60 | 46 | 44 | 49 | 55 | 30 |
| 10 | 93 | 88 | 96 | 82 | 66 | 68 | 58 | 62 | 45 | 52 | 49 | 36 | 34 |
| 11 | 109 | 105 | 113 | 98 | 89 | 89 | 80 | 69 | 48 | 59 | 60 | 55 | 40 |
| 12 | 100 | 104 | 134 | 111 | 127 | 104 | 98 | 80 | 65 | 85 | 80 | 72 | 45 |
| 13 | 155 | 144 | 190 | 156 | 162 | 127 | 156 | 163 | 113 | 132 | 114 | 107 | 72 |
| 14 | 200 | 204 | 227 | 208 | 227 | 185 | 167 | 180 | 144 | 181 | 156 | 159 | 87 |
| 15 | 200 | 191 | 240 | 224 | 232 | 195 | 191 | 199 | 177 | 189 | 194 | 171 | 117 |
| 16 | 185 | 181 | 218 | 217 | 231 | 200 | 198 | 217 | 180 | 198 | 186 | 195 | 124 |
| 17 | 153 | 174 | 196 | 194 | 188 | 168 | 205 | 189 | 167 | 151 | 163 | 150 | 132 |

Source: Own calculation on data from Statistics Denmark.

**Table S4. Number of Girls Experiencing First Foster Care Placement in Denmark by Age (0-17) and Year (1998-2010).**

|  | Year | | | | | | | | | | | | |
| --- | --- | --- | --- | --- | --- | --- | --- | --- | --- | --- | --- | --- | --- |
| Age | 1998 | 1999 | 2000 | 2001 | 2002 | 2003 | 2004 | 2005 | 2006 | 2007 | 2008 | 2009 | 2010 |
| 0 | 74 | 91 | 82 | 82 | 76 | 65 | 66 | 75 | 73 | 85 | 92 | 100 | 64 |
| 1 | 50 | 44 | 43 | 31 | 38 | 30 | 35 | 25 | 23 | 27 | 29 | 30 | 16 |
| 2 | 52 | 45 | 42 | 33 | 33 | 20 | 20 | 27 | 16 | 16 | 21 | 23 | 20 |
| 3 | 65 | 45 | 42 | 45 | 29 | 33 | 34 | 33 | 19 | 24 | 32 | 17 | 13 |
| 4 | 45 | 41 | 44 | 32 | 37 | 24 | 30 | 27 | 16 | 21 | 22 | 29 | 17 |
| 5 | 32 | 46 | 35 | 42 | 48 | 22 | 35 | 21 | 28 | 31 | 24 | 15 | 16 |
| 6 | 51 | 53 | 53 | 29 | 38 | 30 | 28 | 28 | 17 | 33 | 23 | 24 | 20 |
| 7 | 46 | 58 | 40 | 55 | 41 | 28 | 41 | 33 | 31 | 27 | 37 | 26 | 25 |
| 8 | 48 | 53 | 44 | 46 | 45 | 30 | 39 | 38 | 30 | 27 | 29 | 30 | 21 |
| 9 | 38 | 47 | 51 | 39 | 45 | 35 | 34 | 29 | 28 | 32 | 28 | 21 | 15 |
| 10 | 54 | 68 | 50 | 54 | 44 | 41 | 37 | 38 | 36 | 27 | 31 | 29 | 19 |
| 11 | 57 | 67 | 59 | 45 | 58 | 45 | 54 | 40 | 40 | 48 | 48 | 37 | 29 |
| 12 | 63 | 74 | 83 | 76 | 61 | 52 | 58 | 71 | 72 | 72 | 58 | 60 | 25 |
| 13 | 101 | 100 | 126 | 131 | 112 | 136 | 121 | 117 | 115 | 111 | 130 | 102 | 76 |
| 14 | 148 | 171 | 199 | 173 | 185 | 172 | 189 | 175 | 157 | 164 | 186 | 170 | 102 |
| 15 | 198 | 178 | 242 | 211 | 204 | 169 | 187 | 196 | 181 | 196 | 201 | 170 | 102 |
| 16 | 207 | 202 | 211 | 258 | 228 | 194 | 217 | 207 | 176 | 191 | 188 | 170 | 109 |
| 17 | 164 | 190 | 206 | 211 | 205 | 187 | 196 | 190 | 136 | 172 | 148 | 137 | 109 |

Source: Own calculation on data from Statistics Denmark.
